# Virgin olive oil ameliorates deltamethrin-induced nephrotoxicity in mice: A biochemical and immunohistochemical assessment

**DOI:** 10.1016/j.toxrep.2016.07.004

**Published:** 2016-07-30

**Authors:** Ali Reza khalatbary, Hassan Ahmadvand, Davood Nasiry Zarrin Ghabaee, Abbasali Karimpour Malekshah, Azam Navazesh

**Affiliations:** a Molecular and Cell Biology Research Center, Department of Anatomy, Faculty of Medicine, Mazandaran University of Medical Sciences, Sari, Iran; b Department of Biochemistry, Faculty of Medicine, Lorestan University of Medical Sciences, Khorramabad, Iran

**Keywords:** Deltamethrin, Virgin olive oil, Antioxidant, Apoptosis, Inflammation, Nephrotoxicity

## Abstract

**Objective:**

A major class of synthetic pyrethroid insecticide, deltamethrin (DM), can elicit pathophysiological effects through oxidative stress in non-targeted organisms such as mammals. There is accumulating evidence that virgin olive oil (VOO), a rich source of polyphenolic components, have anti-oxidant, anti-inflammatory, and anti-apoptotic properties. This study aimed to determine the protective and ameliorative effects of VOO against DM-induced nephrotoxicity.

**Methods & materials:**

Mice were randomly divided into four equal groups: DM group, DM plus VOO group, VOO group, and vehicle group. Five weeks after gavaging, kidney samples were taken for biochemical assessment of malondialdehyde (MDA), glutathione (GSH) and catalase (CAT), and for immunohistochemical assessment of caspase-3, cyclooxygenase-2 (cox-2) and poly (ADP-ribose) polymerase (PARP).

**Results:**

The MDA level in kidney was increased in the DM group, which was significantly decreased after VOO administration in the DM plus VOO group. The GSH level and CAT activiy in kidney were decreased in the DM group, which were significantly increased after VOO administration in the DM plus VOO group. Greater expression of caspase-3, cox-2, and PARP could be detected in the DM group, which was significantly attenuated in the DM plus VOO group. Also, the histopathological changes which were detected in the DM group attenuated after VOO consumption.

**Conclusion:**

Virgin olive oil exerted protective effects against deltamethrin-induced nephrotoxicity, which might be associated with its anti-apoptotic, anti-inflammatory, and anti-oxidative properties.

## 1 Introduction

Comparatively safe insecticides, pyrethroids, have been classified as type I or type II based upon their chemical structure and clinical manifestations of acute exposure [1]. Deltamethrin is a type II synthetic pyrethroid insecticide with relatively low mammalian toxicity which is used worldwide as a major class of insecticides in agriculture [2]. Studies have shown that deltamethrin is readily absorbed through contaminated water and food [2], and it is bioavailable in feces and urine [3]. In spite of its rapid metabolism and low toxicity, numerous studies documented that chronic exposure to deltamethrin have some of side effects in non-targeted organisms, including neurotoxicity [4], genotoxicity [5], haemolysis [6], reproductive damages [7], pulmonary disorders [8], and hepatotoxicity [9]. Recently, it was also reported that exposure to deltamethrin can elicit nephrotoxicity and cause degenerative changes in kidney tissue [10], [11]. Production of free radicals, induction of lipid peroxidation, disturbance of the total body's antioxidant capacity, inflammation, and apoptosis account the main mechanisms for the deltamethrin toxicity in non-targeted organisms [10], [12]. Therefore, it seems that the use of antioxidant supplements is essential to subside the side effects. Within the previous decades, a rapidly growing number of natural polyphenols, secondary metabolites of plants, with anti-oxidant, anti-inflammatory, and anti-apoptotic effects have been described. One of the main sources of these molecules is olive oil. Olive oil is a rich source of polyphenolic components which have many beneficial health effects in human [13]. There is accumulating evidence that attributed the beneficial effects of olive oil to a variety of biological activities such as free radical scavenging actions which is mediated by chelating of metal ions and providing of hydroxyl group for quenching and neutralization of free radicals [14], [15], anti-inflammatory potency which is mediated by attenuation of anti-inflammatory mediators, and anti-apoptotic properties which is mediated by inhibition of proapoptotic and induction of anti-apoptotic proteins [16], [17]. Meanwhile, olive oil consumption increases total plasma antioxidant activity [18].

Accordingly, in the present study, we investigated the protective effects of virgin olive oil consumption against deltamethrin induced-nephrotoxicity.

## 2 Methods & materials

### 2.1 Virgin olive oil

Virgin olive oil purchased from Giah Essence Phytopharm Co. (Iran). The Chemical composition of the oil is shown in the table below (taken from the Company's website, www.giahessence.com).

**Table:** 

Major phenolics and fatty acids	
Phenolics (mg/kg)	Hydroxytyrosol (0.48 ± 0.02)
	Tyrosol (0.96 ± 0.30)
	Vanilic acid (1.01 ± 0.18)
	Cinamic acid (0.92 ± 0.41)
Fatty acid (%)	Linoleic acid (3.69 ± 0.16)
	Linolenic acid (0.43 ± 0.03)
	Stearic acid (2.24 ± 0.29)
	Oleic acid (75.17 ± 2.66)
	Palmitic acid (16.80 ± 2.55)
	Palmitoloeic acid (1.35 ± 0.37)

### 2.2 Animals

Adult male mice (25 ± 3.0 g) were used (laboratory animal research center, Sari, Iran) in this study. They were kept in the laboratory under constant conditions of temperature (23 ± 2 °C) and light/dark cycle (12 h/12 h) for at least one week before and through the experimental work. All procedures were done according to the guidelines of the university's animal care codes (code; Amums.rec.1392.135) to minimize the animal's suffering and were fed a standard mice chow and drinking water ad libitum throughout the study period.

### 2.3 Grouping

The animals were randomly allocated in four groups, each containing 5 mice: (1) Deltamethrin (DM) treated group, which received DM (Sigma-Aldrich, Germany) diluted in dimethyl sulfoxide (Sigma-Aldrich, Germany) (at 5 mg/kg/day for a period of five weeks by gavages [19]; (2) DM plus virgin olive oil (VOO) treated group, which received 0.4 mL of VOO by gavages for five weeks after 2 h of DM administration [20]; (3) VOO treated group, which received 0.4 mL of VOO by gavages for five weeks; (4) Vehicle group, which received 2.5% diluted DMSO by gavages for five weeks. At the end of the experiment, all mice were euthanized with an injection of sodium pentobarbital and then kidneys were harvested for biochemical, histopathological, and immunohistochemical assessments.

### 2.4 Biochemistry

The obtained samples (right kidney) were thoroughly cleaned of blood, and then were immediately frozen and stored in a −80 °C freezer for assays of tissue malondialdehyde (MDA) levels as a product of lipid peroxidation [21], glutathione (GSH) levels [22], and catalase (CAT) activities [23]. The absorbance of the supernatant was measured by spectrophotometery. MDA and GSH levels were expressed as micromoles per milligram of protein. CAT activity was expressed as unit per milligram of protein.

### 2.5 Histopathology

The obtained samples (3-mm thick sections of the left kidney) were thoroughly cleaned of blood, and then were immediately fixed in 10% (w/v) PBS-buffered formaldehyde and embedded in paraffin. Five-micrometer serial sections were prepared from the paraffin-embedded blocks using microtome. For histopathological assessment, some tissue sections were deparaffinized with xylene, stained with periodic acid-Schiff (PAS) (Sigma-Aldrich, Germany), and studied by light microscopy (DME; Leica Microsystems Inc., Buffalo, NY, USA) to assess the histopathological changes. All the histological studies were performed in a blinded fashion.

### 2.6 Immunohistochemistry

For immunohistochemistry, sections were incubated in normal serum (in order to block non-specific site), and then with anti-caspase 3 rabbit polyclonal antibody (1:100 in PBS, v/v, Abcam), anti-COX 2 rabbit polyclonal antibody (1:100 in PBS, v/v, Abcam) and anti-PARP rabbit polyclonal antibody (1:100 in PBS, v/v, Abcam) overnight at 4 °C. Sections were washed with PBS and then incubated with secondary antibody conjugated with horseradish peroxidase (goat anti-rabbit IgG, Abcam) for 2 h and detected by diaminobenzidine tetrahydrochloride for 5 min. After wards, they were dehydrated and mounted. For negative controls, primary antibodies were omitted. For quantitative analysis at percent of total tissue area, immunohistochemical photographs (n = 5 photos from each samples collected from all mice in each experimental group) were assessed by densitometry using MacBiophotonics ImageJ 1.41a software on an ASUS personal computer. Data are expressed as a percentage of total tissue area.

### 2.7 Statistical analysis

Statistical analysis was carried out in SPSS (Version 15, Chicago, IL, USA). Results were presented as mean values (±SD). The K-S test was used in order to evaluate the normality of the data. Also, the Tukey׳s multiple comparison tests and the analysis of the variance were used to compare each two groups and data among the groups, respectively. A value of p < 0.05 was considered significant.

## 3 Results

### 3.1 Biochemical analysis

Malondialdehyde (MDA) levels for all groups at the end of the experiment are shown in Table 1. The MDA levels were 46.47 ± 0.66 for the Vehicle group, 40.11 ± 0.14 for the VOO group, 57.68 ± 0.45 for the DM group, and 48.24 ± 0.33 for the DM plus VOO group. Administration of deltamethrin in the DM group produced a significant elevation (p < 0.05) in lipid peroxidation level compared to other groups. The MDA levels in the DM plus VOO group were significantly lower than that in the DM group (p < 0.05). The differences between DM plus VOO and vehicle were not significant (p > 0.05), while between DM plus VOO and VOO were significant (p < 0.05).

**Table 1:** Effect of virgin olive oil (VOO) on biochemical markers of mice kidneys affected by deltamethrin nephrotoxicity. Columns that have no superscript common are significantly different from each other (p < 0.05).

Experimental Groups	MDA	GSH	CAT
	μmol/mg-protein	μmol/mg-protein	Unit/mg-protein
Vehicle	46.47 ± 0.66^a^	7.74 ± 0.50^a^	399.30 ± 26.47^a^
VOO	40.11 ± 0.14^b^	8.09 ± 0.17^a^	407.10 ± 0.01^a^
DM	57.68 ± 0.45^c^	6.03 ± 0.01^b^	253.90 ± 8.76^b^
DM + VOO	48.24 ± 0.33^a^	7.53 ± 0.02^a^	336.80 ± 0.01^a^

Glutation (GSH) levels for all groups at the end of the experiment are shown in Table 1. The GSH levels were 7.74 ± 0.50 for the Vehicle group, 8.09 ± 0.17 for the VOO group, 6.03 ± 0.01 for the DM group, and 7.53 ± 0.02 for the DM plus VOO group. Administration of deltamethrin in the DM group produced a significant (p < 0.05) decrease in GSH level compared to the other groups. We found significantly (p < 0.05) increased the GSH levels in DM plus VOO group compared to DM group, while the differences between DM plus VOO, vehicle, and VOO were not significant (p > 0.05).

Catalase (CAT) activity levels for all groups at the end of the experiment are shown in Table 1. The CAT activity was 399.30 ± 26.47 for the Vehicle group, 407.10 ± 0.01 for the VOO group, 253.90 ± 8.76 for the DM group, and 336.80 ± 0.01 for the DM plus VOO group. Administration of deltamethrin in the DM group produced a significant (p < 0.05) decrease in CAT activity compared to the other groups. We found significantly (p < 0.05) increased the CAT activity in DM plus VOO group compared to DM group, while the differences between DM plus VOO, vehicle, and VOO were not significant (p > 0.05).

### 3.2 Histopathologic changes

**Fig. 1:**
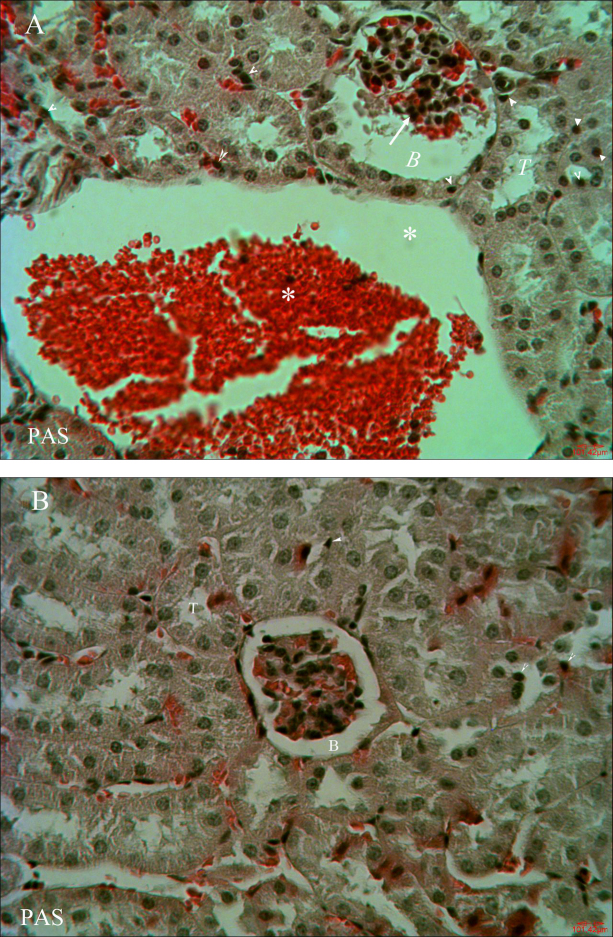
Photomicrographs of kidney sections of DM group and DM plus VOO group (stained with PAS, × 400). Sections of kidney of DM treated rats (1A, 1B) showing enlarged and congested renal vein (asterisks), swelling of renal tubules (T), atrophied glomeruli (arrow) and dilatation of the bowman capsule (B), and pyknotic nuclei of renal epithelium (arrowheads). Sections of kidney of DM + VOO group (1C) showing mild dilation of the bowman capsule (B) and renal tubules (T), and focal nuclear pyknosis of renal epithelium (arrowheads).

Results of histopathological examination are shown in Fig. 1. Treating animals with deltamethrin in DM group revealed many histological alternations (Fig. 1A). The renal veins were enlarged and congested with blood, the renal tubules showed wide lumen, and the glomeruli were atrophied. Meanwhile, cystic dilatations of the bowman capsule and pyknotic nuclei of renal epithelium were observed. Treatment with virgin olive oil in DM plus VOO animals ameliorated these histopathological alternations, so that only focal nuclear pyknosis of renal epithelium and mild dilation of the bowman capsule and renal tubules were observed (Fig. 1B). There were no histopathological changes observed in Vehicle or VOO groups.

### 3.3 Immunohistochemical assessment

**Fig. 2:**
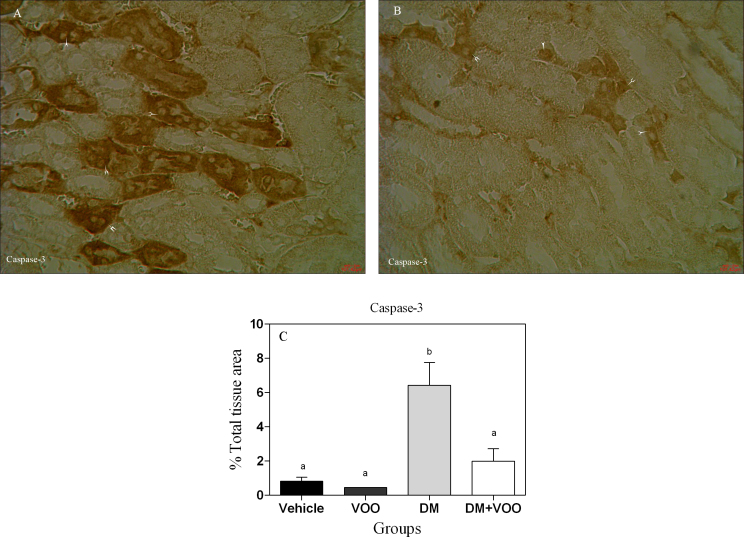
Light Photomicrographs show immunohistochemical expression of caspase-3 in DM (2A) and DM plus VOO (2B) groups (magnification, × 400). The positive staining of caspase-3 is presented by a brown color of cytoplasm (arrowheads). Densitometry analysis of immunohistochemical photomicrographs for caspase-3 was assessed (2C). Data are expressed as a percentage of total tissue area. Columns that have no superscript common are significantly different from each other (p < 0.05).

**Fig. 3:**
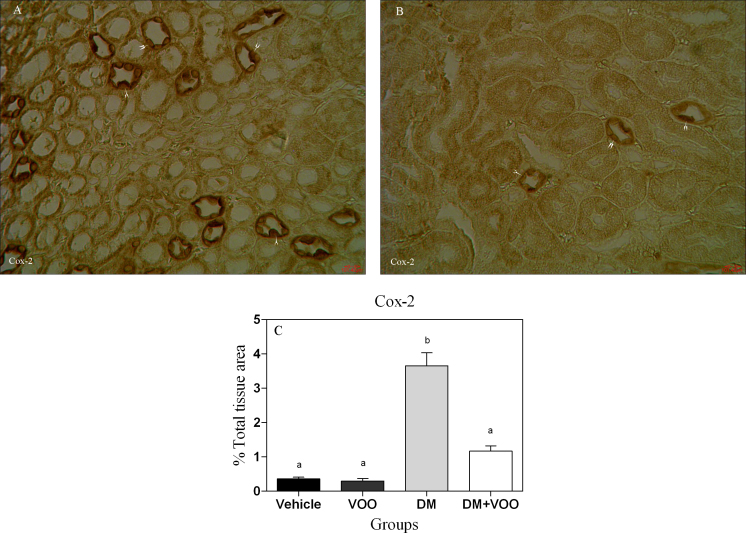
Light Photomicrographs show immunohistochemical expression of cox-2 in DM (3A) and DM plus VOO (3B) groups (magnification, × 400). The positive staining of cox-2 is presented by a brown color of cytoplasm (arrowheads). Densitometry analysis of immunohistochemical photomicrographs for cox-2 was assessed (3C). Data are expressed as a percentage of total tissue area. Columns that have no superscript common are significantly different from each other (p < 0.05).

**Fig. 4:**
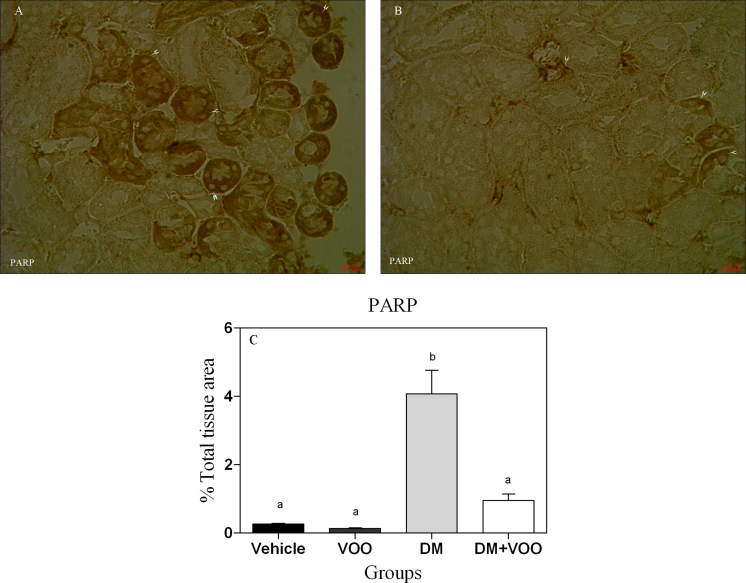
Light Photomicrographs show immunohistochemical expression of PARP in DM (4A) and DM plus VOO (4B) groups (magnification, × 400). The positive staining of PARP is presented by a brown color of cytoplasm (arrowheads). Densitometry analysis of immunohistochemical photomicrographs for PARP was assessed (4C). Data are expressed as a percentage of total tissue area. Columns that have no superscript common are significantly different from each other (p < 0.05).

Fig. 2, Fig. 3, Fig. 4 show the immunohistochemical staining of caspase-3, cox-2, and PARP, respectively. Administration of deltamethrin in DM group increased the expression of caspase-3 (Fig. 2A), cox-2 (Fig. 3A), and PARP (Fig. 4A). While, treatment with virgin olive oil in DM plus VOO group reduced the degree of positive staining for caspase-3 (Fig. 2B), cox-2 (Fig. 3B), and PARP (Fig. 4B) compared to DM group. The histograms of the quantitative analysis of caspase-3, cox-2, and PARP positive staining in the experimental groups are shown in Figs. 2C, 3C, and 4C, respectively.

## 4 Discussion

The main findings of the current study showed that administration of virgin olive oil attenuates histopathological changes, apoptosis, inflammation, and lipid peroxidation. Meanwhile, it improves antioxidant capacity in kidney tissue against deltamethrin-induced nephrotoxicity.

Despite its low mammalian toxicity and worldwide use in agriculture, chronic exposures to deltamethrin have some of undesirable effects on different organs, including the kidney. In this regard, studies have shown that deltamethrin administration increased significantly kidney MDA content, as an indicator of lipid peroxidation, in rats compared with the control group [9], [24]. Lipid peroxidation is an important pathologic event, polyunsaturated fatty acids' breakdown, which is induced by free radicals [25]. Our results showed that elevated MDA levels attenuated significantly after administration of virgin olive oil in the treatment group. Olive oil contains a large amount of molecules such as several different combinations of phenolic antioxidants, free radical scavengers well known that neutralizes the toxic species and sometimes even prevent the early stages of their formation [26], [27]. In this regard, some studies documented the protection of kidney tissue lipid peroxidation with virgin olive oil against mercuric chloride-induced nephrotoxicity [28], with olive leaf extract on gentamicin-induced nephrotoxicity [29], and with hydroxytyrosol as a well-known antioxidant polyphenol from olive oil on cyclosporine nephrotoxicity [30] compared to control groups. Endogenous antioxidants prevent cellular oxidative damage caused by free radicals. Studies have shown that the total antioxidant capacity, glutathione levels, catalase and superoxide dismutase activities in kidney tissues were significantly decreased after deltamethrin administration compared to control groups [11], [31], [32]. Our results showed that administration of deltamethrin decreased glutathione levels and catalase activities in the kidney tissues, meanwhile the decrease somewhat attenuated after administration of virgin olive oil in the treatment group. In this regard, it was documented that virgin olive oil and phenolics such as hydroxytyrosol restored the antioxidant status in kidney after cyclosporine- and mercuric chloride-induced nephrotoxicity in rats [28], [30]. In another study, it was founded that olive leaf extract ameliorates reduced renal glutathione peroxidase, catalase, and superoxide dismutase after cyclosporine-induced nephrotoxicity in rats [33].

Apoptosis is a key mechanism of degenerative diseases which is triggered by some factors such as toxins. In vivo and in vitro studies revealed that exposure to deltamethrin significantly affected the cell survival and induced apoptosis in thymic cells [34], neuronal cell [35], hepatocytes [36], germ cells [37], splenocytes [38], and PC12 cells [39]. Also, recently it was documented that deltamethrin killed MDCK renal tubular cells by Ca^2D^-independent apoptosis [10]. Our immunohistochemical results showed that administration of deltamethrin considerably increased the expression of caspase-3 which plays a critical role in apoptosis, and PARP which is activated by strand break in DNA and resulted ultimately to cell death. On the other hand, our results showed that these upregulations significantly decreased after oral virgin olive oil administration. It is well known that one of the protective mechanisms of dietary virgin olive oil and its phenolics is its effects on the apoptotic process. In this regard, Potocnjak et al. documented that oleuropein, a main olive oil phenolic compound, exerted protective effects against cisplatin-induced apoptosis through attenuation of P53, Bax and caspase-3 expression in kidney [40]. On the other hand, one of the main mechanisms for the deltamethrin toxicity in non-targeted organisms is inflammation. Recently, studies documented that deltamethrin significantly increased the tumor necrosis factor-α (TNF- α) and caused degenerative changes in kidney tissue after oral administration [41], [42]. Our immunohistochemical results showed that administration of deltamethrin considerably increased the expression of cyclooxygenase-2 (cox-2), an enzyme involved in the inflammation, and degenerative changes. Meanwhile, these upregulations and degenerations decreased after virgin olive oil consumption. In this regard, other studies have shown that administration of olive oil and its polyphenols markedly reduced elevated inflammatory indicators such as tumor necrosis factor-alpha and cyclooxygenase-2 after nephrotoxicity [28], [40].

## 5 Conclusion

In sum, our results support that virgin olive oil, a good source of phytochemicals; can markedly attenuate the indicators of the deltamethrin-induced nephrotoxicity; so it can be recommended as a dietary supplement to reduce the side effects of synthetic pyrethroid insecticides.
